# Supplementary material for: Direct observation of the conformational states of PIEZO1
Source: Nature. 2023 Aug 16;620(7976):1117–25. doi: 10.1038/s41586-023-06427-4 (PMC10468401; doi:10.1038/s41586-023-06427-4)
Supplement: Supplementary file 1 — PIEZO protein sequences used for binding energy calculations. [file 41586_2023_6427_MOESM1_ESM.pdf]

nature portfolio
https://doi.org/10.1038/s41586-023-06427-4

Supplementary information

Direct observation of the conformational states of PIEZO1

In the format provided by the
authors and unedited

Supplementary Notes

PIEZO protein sequences used for binding energy calculations:

PIEZO2 6KG7
>Repeat_A
QIYVPIRQFFYDLIHPDYSAVTDVYVLMFLADTVDFIIIVFGFWAFGVPGPFLVMVLIQFGTMVVDRALYL
RKTVLGKVIFQVILVFGIHFWMFFILPGVTERKFSQNLVAQLWYFVKCVYFGLSAYQIRCGYPTRV
>Repeat_B
FLLLFYAMYNTLVARSEMVCYFVIILNHMTSASIITLLLPILIFLWAMLSVPRPSRRFWMMAIVYTEVAIV
VKYFFQFGNIIGVEKKEGYVLYDLIQLLALFFHRSILKCHGLWDE
>Repeat_C
ICMNLDAASFSQHNPVPDFIHCRSYLDMSKVIIFSYLFWFVLTIIFITGTTRISIFCMGYLVACFYFLLFGGD
LLLKPIKSILRYWDWLIAYNVFVITMKNILSIAGIIWDSICFAFLLLQRRVFMSY
>Repeat_D
SKTIFHDITRLHLDDGLINCAKYFVNYFFYKFGLETCFLMSVNVIGQRMDFYAMIHACWLIGVLYRRRRK
AIAEVWPKYCCFLACIITFQYFVCIGIPPAPCRDYPWRFKGAYFNDNIIKWLYFPDFIVRPNPVFLVYDFML
LLCASLQRQIFEDENKAAVRIMAGDNVE
>Repeat_E
TVLFLKFLEYFHKLQVFMWWILELHIIKIVSSYIIWVTVKEVSLFNYVFLISWAFALPYAKLRRAASSVCT
VWTCVIIVCKMLYQLQTIKPENFSVNCSLPNENQTNIPLHELNKSLLYSAPVDPTEWVGLRKSSPLLVYLR
NNLLMLAILAFEVTVYRHQEYYRGRNNLTAPV
>Repeat_F
IMKVLGNLVVALFIKYWIYVCGGMFFFVSFEGKIVMYKIIYMVLFLFCVALYQVHYEWWRKILKYFWM
SVVIYTMLVLIFIYTYQFENFPGLWQNMTGLKKEKLEDLGLKQFTVAELFTRIFIPTSFLLVCILHLHYFHD
RFLELTDLK
>Repeat_G
AVFQFIMKQSYICALIAMMAWSITYHSWLTFVLLIWSCTLWMIRNRRKYAMISSPFMVVYANLLLVLQY
IWSFELPEIKKVPGFLEKKEPGELASKILFTITFWLLLRQHLTEQKALREKEALLS
>Repeat_H
LRRFASVASKLKEFIGNMITTAGKVVVTILLGSSGMMLPSLTSAVYFFVFLGLCTWWSWCRTFDPLLFGC
LCVLLAIFTAGHLIGLYLYQFQFFQEAVPPNDYYWYHHANPILLLVMYYTLATLIRIWLQE
>Repeat_I
GLIFRLLLPICLAVACAFRYNGLSFVYLIYLLLIPLFSEPTKATMQGHTGRLLQSLCITSLSFLLLHIIFHIVF
VPDIGMFIASLTIWLVCRT
>Central_domain
YFLHVVADIKASQILASRGAELFQATIVKAVKARIEEEKKSMDQLKRQMDRIKARQQKYKKGKERVDH
ASMVRSGDYYLFETDSEEFTWVLFLATVDSFTTWLNSISREHIDISTVLRIERCMLTREIKKGNVPTRESIH
MYYQNHLTASDLLMSKMFHDDELEESEKFYVDQPRLGNFLTKSYNYVNLFLFQGFRLVPFLTELRAVM
DWVWTDTTLSLSSWICVEDIYAHIFILKCWRESEKRYPQPRGQKKKKAVKYGMGGMIIVLLICIVWFPLL
FGVINQPLDVSVTITLGGYQPIFTMSAQQSQLKVMDNSKYNEFLKSFGPNSGAMQFLENYEREDVTVAE
LEGNSNSLWTISPPSKQKMIQELTDPNSCFSVVFSWSIQRNMTLGAKAEIATDKLSFPLAVATRNSIAKMI
AGNDTESSNTPVTIEKIYPYYVKAPSDSNSKPIKQLLSENNFMNITIILFRDNVTKSNSEWWVLNLTGSRIF
NQGSQALELVVFNDKVSPPSGIMGLYASVVLVIGKFVREFFSGISHSIMFEELPNVDRILKLCTDIFLVRET
GELELEEDLYAKLIFLYRSPETMIKWTREKTN

PIEZO2 6KG7 – TMDs only
>Repeat_A
QIYVPIRQFFYDLIHPDYSAVTDVYVLMFLADTVDFIIIVFGFWAFGVPGPFLVMVLIQFGTMVVDRALYL
RKTVLGKVIFQVILVFGIHFWMFFILPGVTERKFSQNLVAQLWYFVKCVYFGLSAYQIRCGYP

>Repeat_B
RSEMVCYFVIILNHMTSASIITLLLPILIFLWAMLSVPRPSRRFWMMAIVYTEVAIVVKYFFQFGNIIGVEK
KEGYVLYDLIQLLALFFHRSILKCHGLWDE
>Repeat_C
VIIFSYLFWFVLTIIFITGTTRISIFCMGYLVACFYFLLFGGDLLLKPIKSILRYWDWLIAYNVFVITMKNILSI
AGIIWDSICFAFLLLQRRVFMSYYFL
>Repeat_D
FYKFGLETCFLMSVNVIGQRMDFYAMIHACWLIGVLYRRRRKAIAEVWPKYCCFLACIITFQYFVCVFLV
YDFMLLLCASLQRQIFEDE
>Repeat_E
LHIIKIVSSYIIWVTVKEVSLFNYVFLISWAFALPYAKLRRAASSVCTVWTCVIIVCKMLPLLVYLRNNLL
MLAILAFEVTVYRHQEY
>Repeat_F
IMKVLGNLVVALFIKYWIYVCGGMFFFVSFEGKIVMYKIIYMVLFLFCVALYQVHYEWWRKILKYFWM
SVVIYTMLVLIFIYTYQAELFTRIFIPTSFLLVCILHLHYFHDRFLELTDLK
>Repeat_G
AVFQFIMKQSYICALIAMMAWSITYHSWLTFVLLIWSCTLWMIRNRRKYAMISSPFMVVYANLLLVLQY
IWSGELASKILFTITFWLLLRQHLTEQKALREKEALL
>Repeat_H
LRRFASVASKLKEFIGNMITTAGKVVVTILLGSSGMMLPSLTSAVYFFVFLGLCTWWSWCRTFDPLLFGC
LCVLLAIFTAGHLIGLYLYWYHHANPILLLVMYYTLATLIRIWLQE
>Repeat_I
GLIFRLLLPICLAVACAFRYNGLSFVYLIYLLLIPLFSEPTKATMQGHTGRLLQSLCITSLSFLLLHIIFHIVF
VPDIGMFIASLTIWLVCRT

PIEZO1 AlphaFold II EJ2F22
>Repeat_A
SFYQPLQRFFHDILHTKYRAATDVYALMFLADIVDIIIIIFGFWAFGKHSAATDIASSLSDDQVPQAFLFML
LVQFGTMVIDRALYLRKTVLGKLAFQVVLVVAIHIWMFFILPAVTERMFSQNAVAQLWYFVKCIYFALS
AYQIRCGYPTRI
>Repeat_B
TLRLLRAGYQCVAAHSELLCYFIIILNHMVTASAASLVLPVLVFLWAMLTIPRPSKRFWMTAIVFTEVMV
VTKYLFQFGFFPWNSYVVLRRYENKPYFPPRILGLEKTDSYIKYDLVQLMALFFHRSQLLCYGLWD
>Repeat_C
LEPLRGEPNPIPNFIHCRSYLDMLKVAVFRYLFWLVLVVVFVAGATRISIFGLGYLLACFYLLLFGTTLLQ
KDTRAQLVLWDCLILYNVTVIISKNMLSLLSCVFVEQMQSNFCWVIQLFSLVCTVKGYYDPKEMMTRD
RDCLLPVEEAGIIWDSICFFFLLLQRRIFLSHY
>Repeat_D
AQAVCADGTRQRLDQDLLSCLKYFINFFFYKFGLEICFLMAVNVIGQRMNFMVILHGCWLVAILTRRRR
EAIARLWPNYCLFLTLFLLYQYLLCLGMPPALCIDYPWRWSKAIPMNSALIKWLYLPDFFRAPNSTNLIS
DFLLLLCASQQWQVFSAERTEEWQRMAGINTDH
>Repeat_E
LDLAASFSAVLTRIQVFVRRLLELHVFKLVALYTVWVALKEVSVMNLLLVVLWAFALPYPRFRPMASCL
STVWTCIIIVCKMLYQLKIVNPHEYSSNCTEPFPNNTNLQPLEINQSLLYRGPVDPANWFGVRKGYPNLG
YIQNHLQILLLLVFEAVVYRRQEHYRRQHQQAPLP
>Repeat_F
TQTQTLLRSLGELVTGIYVKYWIYVCAGMFIVVSFAGRLVVYKIVYMFLFLLCLTLFQVYYTLWRKLLR
VFWWLVVAYTMLVLIAVYTFQFQDFPTYWRNLTGFTDEQLGDLGLEQFSVSELFSSILIPGFFLLACILQL
HYFHRPFMQLTDLEH
>Repeat_G

PLHGLGHLIMDQSYVCALIAMMVWSIMYHSWLTFVLLLWACLIWTVRSRHQLAMLCSPCILLYGLTLC
CLRYVWAMELPELPTTLGPVSLHQLGLEHTRYPCLDLGAMLLYLLTFWLLLRQFVKEKLLKKQKVP
>Repeat_H
RRLWLASRFRVTAHWLLMTSGRTLVIVLLALAGIAHPSAFSSIYLVVFLAICTWWSCHFPLSPLGFNTLC
VMVSCFGAGHLICLYCYQTPFIQDMLPPGNIWARLFGLKNFVDLPNYSSPNALVLNTKHAWPIYVSPGIL
LLLYYTATSLLKLHKS
>Repeat_I
MEPHVLGAGLYWLLLPCTLLAASLLRFNALSLVYLLFLLLLPWLPGPSRHSIPGHTGRLLRALLCLSLLFL
VAHLAFQICLHTVPHLDQFLGQNGSLWVKVSQHIGVTRLDLKDIFNTTRLVAPDLGVLLASSLCLGLCGR
>Central_domain
YFLHVVADIKASQILASRGAELFQATIVKAVKARIEEEKKSMDQLKRQMDRIKARQQKYKKGKERVDH
ASMVRSGDYYLFETDSEEFTWVLFLATVDSFTTWLNSISREHIDISTVLRIERCMLTREIKKGNVPTRESIH
MYYQNHLTASDLLMSKMFHDDELEESEKFYVDQPRLGNFLTKSYNYVNLFLFQGFRLVPFLTELRAVM
DWVWTDTTLSLSSWICVEDIYAHIFILKCWRESEKRYPQPRGQKKKKAVKYGMGGMIIVLLICIVWFPLL
FGVINQPLDVSVTITLGGYQPIFTMSAQQSQLKVMDNSKYNEFLKSFGPNSGAMQFLENYEREDVTVAE
LEGNSNSLWTISPPSKQKMIQELTDPNSCFSVVFSWSIQRNMTLGAKAEIATDKLSFPLAVATRNSIAKMI
AGNDTESSNTPVTIEKIYPYYVKAPSDSNSKPIKQLLSENNFMNITIILFRDNVTKSNSEWWVLNLTGSRIF
NQGSQALELVVFNDKVSPPSGIMGLYASVVLVIGKFVREFFSGISHSIMFEELPNVDRILKLCTDIFLVRET
GELELEEDLYAKLIFLYRSPETMIKWTREKTN

PIEZO1 AlphaFold II EJ2F22 – TMDs only
>Repeat_A
QIYVPIRQFFYDLIHPDYSAVTDVYVLMFLADTVDFIIIVFGFWAFGVPGPFLVMVLIQFGTMVVDRALYL
RKTVLGKVIFQVILVFGIHFWMFFILPGVTERKFSQNLVAQLWYFVKCVYFGLSAYQIRCGYP
>Repeat_B
RSEMVCYFVIILNHMTSASIITLLLPILIFLWAMLSVPRPSRRFWMMAIVYTEVAIVVKYFFQFGNIIGVEK
KEGYVLYDLIQLLALFFHRSILKCHGLWDE
>Repeat_C
VIIFSYLFWFVLTIIFITGTTRISIFCMGYLVACFYFLLFGGDLLLKPIKSILRYWDWLIAYNVFVITMKNILSI
AGIIWDSICFAFLLLQRRVFMSYYFL
>Repeat_D
FYKFGLETCFLMSVNVIGQRMDFYAMIHACWLIGVLYRRRRKAIAEVWPKYCCFLACIITFQYFVCVFLV
YDFMLLLCASLQRQIFEDE
>Repeat_E
LHIIKIVSSYIIWVTVKEVSLFNYVFLISWAFALPYAKLRRAASSVCTVWTCVIIVCKMLPLLVYLRNNLL
MLAILAFEVTVYRHQEY
>Repeat_F
IMKVLGNLVVALFIKYWIYVCGGMFFFVSFEGKIVMYKIIYMVLFLFCVALYQVHYEWWRKILKYFWM
SVVIYTMLVLIFIYTYQAELFTRIFIPTSFLLVCILHLHYFHDRFLELTDLK
>Repeat_G
AVFQFIMKQSYICALIAMMAWSITYHSWLTFVLLIWSCTLWMIRNRRKYAMISSPFMVVYANLLLVLQY
IWSGELASKILFTITFWLLLRQHLTEQKALREKEALL
>Repeat_H
LRRFASVASKLKEFIGNMITTAGKVVVTILLGSSGMMLPSLTSAVYFFVFLGLCTWWSWCRTFDPLLFGC
LCVLLAIFTAGHLIGLYLYWYHHANPILLLVMYYTLATLIRIWLQE
>Repeat_I
GLIFRLLLPICLAVACAFRYNGLSFVYLIYLLLIPLFSEPTKATMQGHTGRLLQSLCITSLSFLLLHIIFHIVF
VPDIGMFIASLTIWLVCRT
