# Supplementary material for: Direct observation of the conformational states of PIEZO1
Source: Nature. 2023 Aug 16;620(7976):1117–25. doi: 10.1038/s41586-023-06427-4 (PMC10468401; doi:10.1038/s41586-023-06427-4)
Supplement: Supplementary file 2 — Reporting Summary [file 41586_2023_6427_MOESM2_ESM.pdf]

nature portfolio

Corresponding author(s): Ardem Patapoutian

Last updated by author(s): June 14, 2023

Reporting Summary

Nature Portfolio wishes to improve the reproducibility of the work that we publish. This form provides structure for consistency and transparency in reporting. For further information on Nature Portfolio policies, see our Editorial Policies and the Editorial Policy Checklist.

Statistics

For all statistical analyses, confirm that the following items are present in the figure legend, table legend, main text, or Methods section.

n/a	Confirmed	
☐	☒	The exact sample size (*n*) for each experimental group/condition, given as a discrete number and unit of measurement
☐	☒	A statement on whether measurements were taken from distinct samples or whether the same sample was measured repeatedly
☐	☒	The statistical test(s) used AND whether they are one- or two-sided<br>*Only common tests should be described solely by name; describe more complex techniques in the Methods section.*
☒	☐	A description of all covariates tested
☒	☐	A description of any assumptions or corrections, such as tests of normality and adjustment for multiple comparisons
☐	☒	A full description of the statistical parameters including central tendency (e.g. means) or other basic estimates (e.g. regression coefficient) AND variation (e.g. standard deviation) or associated estimates of uncertainty (e.g. confidence intervals)
☐	☒	For null hypothesis testing, the test statistic (e.g. *F*, *t*, *r*) with confidence intervals, effect sizes, degrees of freedom and *P* value noted<br>*Give P values as exact values whenever suitable.*
☒	☐	For Bayesian analysis, information on the choice of priors and Markov chain Monte Carlo settings
☒	☐	For hierarchical and complex designs, identification of the appropriate level for tests and full reporting of outcomes
☒	☐	Estimates of effect sizes (e.g. Cohen's *d*, Pearson's *r*), indicating how they were calculated

Our web collection on statistics for biologists contains articles on many of the points above.

Software and code

Policy information about availability of computer code

Data collection	iPALM data was collected with custom LabView software (LabView 2010, National Instruments), as described in Shtengel, G. et al. 2009. MINFLUX data was collected with Imspector software with MINFLUX drivers (version 16.3, Abberior Instruments). Confocal images were collected with NIS-Elements software (version 5.40.01, Nikon). Electrophysiology data was collected with pClamp software (version 10.2 and 10.7, Molecular Devices). PIEZO repeat binding energy calculations were performed using PDBePISA software (version 1.52, https://www.ebi.ac.uk/pdbe/pisa/).
Data analysis	iPALM data pre-processing was performed using PeakSelector software (https://github.com/gleb-shtengel/PeakSelector). iPALM data analysis, particle fusion, and MINFLUX data analysis was performed using custom software written in MATLAB (version R2021b, MathWorks). Confocal and brightfield images were analyzed using Fiji (version 2.30, https://fiji.sc/). DNA sequences were created and analyzed in SnapGene (Version 6.2, Dotmatics). Data visualization and statistical tests were performed with MATLAB 2021 (version R2021b, MathWorks) and Prism (version 9.5, GraphPad). Visualization of localizations in Fig. 2 were performed using ParaView (version 5.10, kitware). Molecular structures were visualized using MolStar viewer (https://molstar.org/viewer/) and Chimera software (version 1.15, UCSF). Graphics were created using Adobe Illustrator (version 2023, Adobe). Code for iPALM data pre-processing wass previously published and available at https://github.com/gleb-shtengel/PeakSelector. Custom MATLAB code for iPALM data analysis is available at https://doi.org/10.5281/zenodo.8017632 and custom MATLAB code for MINFLUX analysis is available at https://github.com/PatapoutianLab/MINFLUX_Piezo_Analysis.

For manuscripts utilizing custom algorithms or software that are central to the research but not yet described in published literature, software must be made available to editors and reviewers. We strongly encourage code deposition in a community repository (e.g. GitHub). See the Nature Portfolio guidelines for submitting code & software for further information.

Data

Policy information about availability of data

All manuscripts must include a data availability statement. This statement should provide the following information, where applicable:

- Accession codes, unique identifiers, or web links for publicly available datasets
- A description of any restrictions on data availability
- For clinical datasets or third party data, please ensure that the statement adheres to our policy

All data including the raw MINFLUX analysis output for each experimental condition are available online as separate Excel files for each figure. Protein structure data was obtained from RCSB Protein Data Bank (PIEZO1 6B3R: https://doi.org/10.2210/pdb6B3R/pdb, PIEZO1 7WLU: https://doi.org/10.2210/pdb7WLU/pdb, and PIEZO2 6KG7: https://doi.org/10.2210/pdb6KG7/pdb) and the AlphaFold Protein Structure Database (PIEZO1: https://alphafold.ebi.ac.uk/entry/E2JF22). Unprocessed data are available from A.P. upon request.

Human research participants

Policy information about studies involving human research participants and Sex and Gender in Research.

Reporting on sex and gender	N/A
Population characteristics	N/A
Recruitment	N/A
Ethics oversight	N/A

Note that full information on the approval of the study protocol must also be provided in the manuscript.

Field-specific reporting

Please select the one below that is the best fit for your research. If you are not sure, read the appropriate sections before making your selection.

☒ Life sciences ☐ Behavioural & social sciences ☐ Ecological, evolutionary & environmental sciences

For a reference copy of the document with all sections, see nature.com/documents/nr-reporting-summary-flat.pdf

Life sciences study design

All studies must disclose on these points even when the disclosure is negative.

Sample size	At least three samples were measured for each condition to account for variability in samples and sample preparation. For MINFLUX imaging, we imaged a minimum of three cells, but in some cases, we continued to acquire data until enough molecules were identified to capture the distribution reflecting the apparent mechanical behavior of the imaged region of the molecule. For iPALM imaging, we determined a sample size of 5 cells based upon the estimated number of molecules needed for a fused superparticle of sufficient resolution, given the number of apparent triple-labeled molecules from the first dataset.
Data exclusions	For imaging of PIEZO1 cell membranes, data outside the plasma membrane was excluded based on either calculated distance to fiducial markers on the coverslip or the distribution of localizations in the z-plane. For MINFLUX imaging of PIEZO1, those identified trimeric molecules which had an inter-blade angle >120° were excluded as these were primarily due to linear "streaks" in the data, likely from freely diffusing molecules which are universally present in various sample types (including samples of different molecules and labels) in our hands.
Replication	At least three biological and experimental replicates were performed for each experiment. All attempts at replication were successful.
Randomization	This study did not allocate experimental units to groups, and so no randomization was required for any experiment reported.
Blinding	Blinding was not relevant to this study because all data was analyzed using the same methods.

Reporting for specific materials, systems and methods

We require information from authors about some types of materials, experimental systems and methods used in many studies. Here, indicate whether each material, system or method listed is relevant to your study. If you are not sure if a list item applies to your research, read the appropriate section before selecting a response.

Materials & experimental systems

n/a	Involved in the study
☒	☐ Antibodies
☐	☒ Eukaryotic cell lines
☒	☐ Palaeontology and archaeology
☒	☐ Animals and other organisms
☒	☐ Clinical data
☒	☐ Dual use research of concern

Methods

n/a	Involved in the study
☒	☐ ChIP-seq
☒	☐ Flow cytometry
☒	☐ MRI-based neuroimaging

Eukaryotic cell lines

Policy information about cell lines and Sex and Gender in Research

Cell line source(s)	Expi293 cells were obtained from ThermoFisher Scientific. Swell1-knockout cells were of the Freestyle HEK293-F cell line, originally obtained from ThermoFisher Scientific, and modified as described in Kefauver, et al. 2018. Piezo1 KO HEK293 cells were generated as described in Lukacs, et al. 2015 and are deposited with ATCC (ATCC CRL-3519).
Authentication	Commercially available cell lines were authenticated by the supplier. Knockout of the genes encoding Swell1 (LRRC8A, LRRC8B, LRRC8D, and LRRC8E) in the Swell1-KO cells were verified in Kefauver, et al. 2018. Successful knock-out of Swell1 genes was determined by PCR genotyping and Sanger sequencing targeted regions for frameshift mutations and verified by mass spectrometry analysis. Knock-out of PIEZO1 was verified using PCR genotyping and Sanger sequencing of PIEZO1 alleles.
Mycoplasma contamination	All cell lines tested negative for mycoplasma contamination using the MycoAlert® Mycoplasma Detection Kit (Lonza).
Commonly misidentified lines (See ICLAC register)	None
